# The prevalence of self-reported underuse of medications due to cost for the elderly: results from seven European urban communities

**DOI:** 10.1186/s12913-015-1089-4

**Published:** 2015-09-26

**Authors:** Aurima Stankuniene, Mindaugas Stankunas, Mark Avery, Jutta Lindert, Rita Mikalauskiene, Maria Gabriella Melchiorre, Francisco Torres-Gonzalez, Elisabeth Ioannidi-Kapolou, Henrique Barros, Arūnas Savickas, Raimondas Radziunas, Joaquim J. F. Soares

**Affiliations:** Department of Drug Technology and Social Pharmacy, Lithuanian University of Health Sciences, Kaunas, Lithuania; Department of Health Management, Lithuanian University of Health Sciences, Kaunas, Lithuania; Health Service Management Department, Centre for Health Innovation, School of Medicine, Griffith University, Gold Coast, Queensland Australia; Department of Public Health, University of Emden, Emden, Germany; Brandeis University, Waltham, USA; Scientific Technological Area, Centre for Socio Economic Research on Ageing, Italian National Institute of Health and Science on Aging (INRCA), Ancona, Italy; Centro de Investigación Biomedica en Red de Salud Mental (CIBERSAM), University of Granada, Granada, Spain; Department of Sociology, National School of Public Health, Athens, Greece; Department of Hygiene and Epidemiology, Faculty of Medicine, University of Porto, Porto, Portugal; Department of Health Sciences, Section of Public Health Science, Mid Sweden University, Sundsvall, Sweden

**Keywords:** Ageing, Accessibility, Medications, Europe, ABUEL

## Abstract

**Background:**

The aim of this study was to evaluate the prevalence of self-reported underuse of medications due to procurement costs amongst older persons from seven European urban communities.

**Methods:**

The data were collected in a cross-sectional study (“ABUEL, Elder abuse: A multinational prevalence survey”) in 2009. Randomly selected people aged 60–84 years (*n* = 4,467) from seven urban communities: Stuttgart (Germany), Athens (Greece), Ancona (Italy), Kaunas (Lithuania), Porto (Portugal), Granada (Spain) and Stockholm (Sweden) were interviewed. Response rate - 45.2 %. Ethical permission was received in each country.

**Results:**

The results indicate that 3.6 % (*n* = 162) of the respondents self-reported refraining from buying prescribed medications due to cost. The highest prevalence of this problem was identified in Lithuania (15.7 %, *n* = 99) and Portugal (4.3 %, *n* = 28). Other countries reported lower percentages of refraining from buying medications (Germany – 2.0 %, Italy – 1.6 %, Sweden – 1.0 %, Greece – 0.6 %, Spain – 0.3 %). Females refrained more often from buying medications than males (2.6 % vs. 4.4 %, *p* < 0.0001). The prevalence of this refraining tended to increase with economic hardship.

**Discussion:**

These differences between countries can be only partly described by the financing of health-care systems. In spite of the presence of cost reimbursement mechanisms, patients need to make co-payments (or in some cases to pay the full price) for prescribed medications. This indicates that the purchasing power of people in 10.1186/s12913-015-1089-4 the particular country can play a major role and be related with the economic situation in the country. Lithuania, which has reported the highest refrain rates, had the lowest gross domestic product (at the time of conducting this study) of all participating countries in the study.

**Conclusions:**

Refraining from buying the prescribed medications due to cost is a problem for women and men in respect to ageing people in Europe. Prevalence varies by country, sex, and economic hardship.

## Background

The use of medicines by elderly people is a growing concern in social pharmacy and beyond [1, 2]. In particular, polypharmacy (the use of multiple medications) is common among the elderly but it may cause many problems such as an increased risk of inappropriate use of medications/drugs, adverse effects and non-adherence. In addition, polypharmacy increases medical costs [3]. Moreover, increasing prices and the proportion of out-of-pocket payments in purchasing necessary pharmaceuticals may lead to situations where some older people refrain from buying prescribed medications [4]. The World Health Organization (WHO) has indicated five broad groupings of potential reasons for medication non-adherence: 1) patient, 2) health condition, 3) therapy, 4) socio-economic and 5) health system–related factors. In particular socio-economic reasons include low health literacy, poor social support and higher medication costs [5]. A review by Perkins (2002) showed that the correlation of poor adherence were as follows: patients’ beliefs about their illness/benefits of treatment, and barriers to treatment such as easy access to treatment, family/social support and perceived costs of treatment [6].

This non-adherence to medication use may cause serious problems to the health status of patients and increased cost to health-care systems in terms of additional hospital admissions. For instance, data from Australia suggest that at least 45,000 older Australians are hospitalized each year because of medication-related problems, representing 20–30 % of unplanned hospital admissions for this age group [7]. Non-adherence due to costs in the elderly, could be of a significant and worrying size problem in European countries. There have been several attempts to measure the size of this problem in European countries and beyond [8]. However, we still lack comparable data on the refraining of purchasing medications due to cost.

In this paper we use data from a cross sectional study in seven European countries (“Elder abuse: a multinational prevalence study – ABUEL” [9] project. One of the objectives of this project was to measure the accessibility and use of health services by older people. The present study is an opportunity to provide reliable data on the issue. Basic characteristics of the healthcare system and pharmaceutical schemes in each country are presented in Table 1 [10–18]. Some papers on use and non-adherence have been published elsewhere [19–22]. However, the referred papers have examined the prevalence of refrain due to any cause [19], or focused on the situation in Lithuania [20–22]. In this paper, we investigate the prevalence of refrain due to financial problems among older persons and to compare it among seven European urban communities.

**Table 1:** Basic characteristics of the healthcare system and pharmaceutical schemes in ABUEL study countries

Indicator	Lithuania	Sweden	Germany	Italy	Spain	Portugal	Greece
Total health expenditure 2011 as % of GDP (PPP$ per capita)^1^	6.66 %	9.62 %	11.28 %	9.18 %	9.62 %	9.46 %	9.28 %
	(1426 PPP$)	(4158 PPP$)	(4617 PPP$)	(3040 PPP$)	(3145 PPP$)	(2400 PPP$)	(2347 PPP$)
Total pharmaceutical expenditure as % of total health 2011^1^	24.9	12.1	14.1	16.2	17.4	17.9	28.5
Public pharmaceutical expenditure as % of total pharmaceutical expenditure 2011^1^	34.2	58.3	75.6	46.6	71.0	55.1	73.7
Predominant health care financing mechanism^2^	Social insurance	Taxes (local)	Social insurance	Taxes (local)	Taxes (local)	Taxes (central)	Social insurance
Reimbursment mechanism for prescribed medicines	NHIF reimbursed 50–100 % of price of selected medicines^3^	The patient has to pay the full cost for prescribed drugs, up to SEK 1100 (€122), after which level the subsidy gradually increase up to a 100 %.^4^	For prescription-only drugs, pharmacists are now paid through a flat-rate payment of €8.35 plus a fixed margin of 3 %. The retail price contains an additional 19 % VAT.^5^	Only Class A medicines are partially reimbursed by the SSN and involve a modest co-payment that varies across regions.^6^ I.e. Veneto Region implies co-payment mechanism from €2 for each packet up to a maximum of €4 for each prescription.^7^	Medical prescriptions funded by the SNS are exempt from co-payment for pensioners and their beneficiaries. nonpensioners and their beneficiaries pay 40 % of the retail price.^8^	There are four groups of medications and reimbursment varies by these categories from 90 % for category A to 5 % for category D. Pensioners have additional reductions from 5 to 15 %.^9^	Insured citizens participate in covering the cost of pharmaceuticals with a co-payment rate set at 25 %. Patients with chronic conditions are exempted from co-payments, while pensioners on lower incomes who are beneficiaries of EKAS pay a co-payment of 10 %. The very poor are entitled to pharmaceuticals provided by public hospitals free of charge.^9^

Surce:^1^ – Ref. 10;^2^ – Ref. 11;^3^–Ref. 12;^4^ – Ref. 13;^5^ – Ref. 14;^6^ – Ref. 15;^7^ - Ref. 16;^8^ - Ref 17,^9^ - Ref 18

*GDP* Gross Domestic Product, *PPP* Purchasing Power Parity, *NHIF* National Health Insurance Fund (Lithuania), *SSN* Italy’s National Health Service, *SNS* Spanish national health system, *EKAS* Social Solidarity Benefit for low-income pensioners

## Methods

Data for this study were collected during the European project ABUEL. The participants consisted of randomly selected women and men from the general population living in urban centres of seven European countries (Germany; Stuttgart; Greece, Athens; Italy, Ancona; Lithuania, Kaunas; Portugal, Porto; Spain, Granada; Sweden, Stockholm), except for Greece where a random route sample was used. Inclusion criteria were: 1) aged 60–84 years; 2) did not suffer from dementia or other cognitive impairments; 3) had a legal status (national citizens or documented migrants); 4) lived in the community or sheltered houses; 5) could read and write in the native languages; and 6) accepted participation to the study. A sample size was calculated based on municipal censuses (women and men aged 60–84 years) and an expected abuse prevalence of 13 % derived from a recent systematic review [23]. The sample size was customized for each country according to the population of individuals aged 60–84 years, with a maximum of 642 individuals in each of the participating countries because of the infinite population assumption. The sample was calculated proportional to age–sex groups in the population in each city. Three sampling approaches were used in ABUEL: 1) registry-based sampling (Germany, Spain, Italy, Lithuania and Sweden); 2) sampling by random route (Greece); and 3) cluster sampling (Portugal). The registry-based sampling was based on the city’s population registries.

The total number of participants amounted to 4,451 (2,576 women, 57.9 %). Response rates in the sampling base varied between countries from 18.9–87.4 %, with a mean of 45.2 % across countries. Response rates for women were 47.1 % and for men 49.3 %, and varied between age groups from 47.0–49.7 %, with a mean of 48 % across age groups.^1^ However, there were no major differences (age and gender) between refusals and non-refusals nor did they differ from the general population in each participating country. The final sample consisted of 4,467 persons (2,559 women, 57.3 %). A more detailed description of sampling, data collection, and study limitations are described in a separate ABUEL methodology paper [24].

The design of the study was cross-sectional. Recruitment and data gathering were performed during January-July, 2009. Written information about the ABUEL study was sent to the eligible individuals’ homes. Trained interviewers telephoned the eligible persons (except in Lithuania) and provided information about the study. Informed consent from participants was obtained before interviewing. Two administration modes were used: (i) face-to-face interviews (Spain, Italy, Greece, Lithuania, Portugal); and (ii) mixed methods, i.e. face-to-face interviews and mailed questionnaires (Germany and Sweden).

Great emphasis was put on confidentiality, anonymity and the rights of older persons. The ethical permissions for the project were given by: *Germany*, Ethikkommission des Landes Baden-Wuerttenberg; *Italy*, Bioethics Advisory Committee of National Institute of Health and Science on Aging, Italian National Institute of Health and Science on Aging; *Lithuania*, The Lithuanian State Data Protection Inspectorate and the Kaunas Regional Bioethics Committee; *Portugal*, Comité de Ética do Hospital de João; *Spain*, Comité de Etica en Investigación de la Universidad de Granada; and *Sweden*, Regional Ethical Committee at Karolinska Institutet. In *Greece*, QED (market research company) conducted the fieldwork under the codes and guidelines of International Chamber of Commerce/European Society for Opinion and Market Research which are similar to ethical provisions in the other participating countries.

The participants completed a standardized questionnaire with various scales and questions [25]. Self-reported refrain from buying prescribed medications due to costs was measured with question: “*What were the reasons for not buying prescribed medications and care”?* (multiple-choice). A specific time frame has not identified. Economic hardship was measured with one question ”*How often are you worried about the daily expenses? (e.g. for buying food)”* in a “*never/quite often/often/always*” format. A participant was defined as having “financial strain” if she/he chose any response other than “never”.

Data were computed, coded and analyzed using the Statistical Package for the Social Sciences for Windows, Version 17.0 (SPSS Inc.). The following statistical analyses were applied: 1) descriptive statistics; 2) logistic regression.

Associations of the reported refrain from buying prescribed medications due to costs and social-economic factors were measured calculating the prevalence of non-adherence. Differences between groups were assessed by using the two-tailed z criteria for categorical variables.

For evaluation of the impact of explanatory variables on analyzed event, (binary dependent variable) *Enter* model of multivariate logistic regression was used. Dependent variable was the reported refrain from buying prescribed medications due to costs (based on answers in the questionnaire). Sex, age, country of residence, living alone, education, economic hardship were used as independent variables. These associations were measured using odds ratio (OR) and calculating the 95 % confidence interval (CI). Differences in results at the *p* < 0.05 level were considered statistically significant.

## Results

Of the 4,467 respondents, 1,908 (42.5 %) were male and 2,559 (57.5 %) were female. The distribution of respondents by age was: 60–64 years (25.2 %), 65–69 years (24.4 %), 70–74 years (21.1 %), 75–79 years (16.1 %) and 80–84 years (12.2 %); and by education: cannot read/write (3.1 %), without any degree (4.2 %), less than primary school (7.5 %), primary school/similar (24.4 %), secondary school/similar (40.0 %), university/similar (19.2 %) and other (1.6 %) (Table 2). A more detailed description of the study sample is presented in a separate paper [26].

**Table 2:** The main socio-demographic characteristics of respondents

Variable	%/n							
	Lithuania	Sweden	Germany	Italy	Spain	Portugal	Greece	All countries
Sex								
Male	35.7/225	46.8/293	47.1/305	43.0/270	42.8/272	39.0/256	44.6/287	42.7/1908
Female	64.3/405	53.2/333	52.9/343	57.0/358	57.2/364	61.0/400	55.4/356	57.3/2559
Age group								
60–64 years	23.2/146	33.9/212	21.7/137	22.5/141	23.3/148	24.5/161	27.8/179	25.2/1124
65–69 years	23.5/148	23.8/149	28.4/184	22.6/142	22.0/140	24.4/160	25.7/165	24.4/1088
70–74 years	23.2/146	16.9/106	23.5/152	20.5/129	22.5/143	21.0/138	22.9/147	21.5/961
74–79 years	19.2/121	13.3/83	16.0/104	18.9/119	17.8/113	17.5/115	14.6/94	16.8/749
80–84 years	11.0/69	12.1/76	11.0/71	15.4/97	14.5/92	12.5/82	9.0/58	12.2/545
Lives alone								
No	75.8/475	66.1/414	67.3/429	86.9/546	82.14/522	78.4/514	73.7/474	75.8/3374
Yes	24.2/152	33.9/212	32.7/208	13.1/82	17.9/114	21.6/142	26.3/169	24.2/1079
Education								
Less than primary	5.6/34	2.3/14	1.3/8	4.3/27	60.0/381	11.4/75	19.0/122	15.1/661
Primary	24.3/147	31.1/193	2.2/13	34.6/217	13.1/83	36.4/239	31.2/200	24.9/1092
Secondary	46.7/283	33.3/207	67.2/405	50.3/316	11.5/73	36.1/237	40.7/261	40.6/1782
University	23.4/142	33.3/207	29.4/177	10.8/68	15.4/98	16.0/105	9.0/58	19.5/855
Economic hardship								
Never	26.8/169	64.0/400	52.5/339	41.4/259	31.8/202	29.7/195	6.4/41	36.0/1605
Quite often	37.8/238	26.4/165	31.7/205	35.8/224	9.9/63	23.0/151	23.5/151	26.8/1197
Often	21.4/135	5.4/34	11.5/74	15.3/96	15.1/96	11.3/74	28.1/181	15.5/690
Always	14.0/88	4.2/26	4.3/28	7.5/47	43.2/275	36.0/236	42.0/270	21.7/970

*n* number of observed persons

**Fig. 1:**
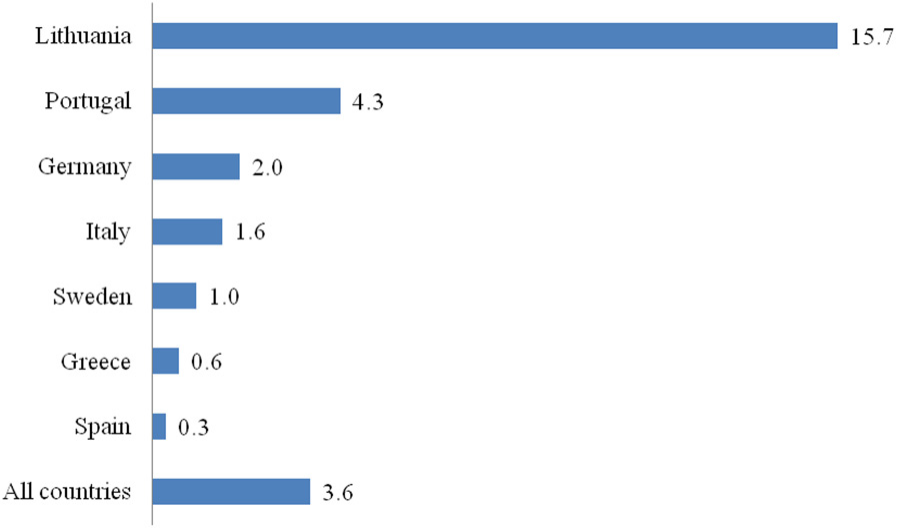
The prevalence (%) of refrain from buying medications by country

The results indicate that 3.6 % of all respondents of the study had refrained from buying the prescribed medications due to cost. Furthermore, prevalence varied by country (Fig. 1). Cost-related non-adherence has been identified in Lithuania 15.7 % (*n* = 99). The other countries reported considerably lower rates. The second highest rate was in Portugal (4.3 %, *n* = 28). The remaining countries had prevalence rates lower than 2 %.

The prevalence of self-reported refrain from buying medications was evaluated by different socio-economic factors in each country and study sample in general (Table 3). It was identified, that the prevalence of refrain due to cost has differed by sex and economic hardship. Females and people experiencing economic problems were more likely not to buy prescribed drugs because of costs. Age, education and living alone were not associated with refrain.

**Table 3:** The prevalence of refrain from buying medications due to cost by different socio-economic variables

Variable	%/n							
	Lithuania	Sweden	Germany	Italy	Spain	Portugal	Greece	All
Sex								
Male (R)	16.0/36	0.7/2	1.6/5	0.7/2	0.0/0	1.6/4	0.0/0	2.6/49
Female	15.6/63	1.2/4	2.3/8	2.2/8	0.5/2	6.0/24**	1.1/4*	4.4/113***
Age group								
60–64 years (R)	20.5/30	2.4/5	2.9/4	2.1/3	0.0/0	1.9/3	1.1/2	4.2/47
65–69 years	16.9/25	0.0/0*	0.5/1	2.1/3	0.7/1	3.8/6	0.6/1	3.4/37
70–74 years	12.3/18	0.9/1	3.9/6	2.3/3	0.0/0	5.8/8	0.7/1	3.9/37
74–79 years	14.0/17	0.0/0*	1.0/1	0.8/1	0.9/1	7.0/8	0.0/0	3.7/28
80–84 years	13.0/9	0.0/0*	1.4/1	0.0/0	0.0/0	3.7/3	0.0/0	2.4/13*
Lives alone								
No (R)	16.4/78	0.7/3	0.9/4	1.5/8	0.2/1	3.3/17	0.6/3	3.4/114
Yes	13.2/20	1.4/3	4.3/ 9*	2.4/2	0.9/1	7.7/11	0.6/1	4.4/47
Education								
Less than primary	26.5/9	7.1/1	0.0/0	0.0/0	0.3/1	17.3/13***	0.0/0	3.6/24
Primary	17.7/26	1.6/3	7.7/1	0.9/2	1.2/1	4.2/10	0.5/1	4.1/45
Secondary	16.3/46	1.0/2	2.2/9	2.2/7	0.0/0	1.3/3	0.8/2	3.8/68
University (R)	11.3/16	0.0/0	1.7/3	1.5/1	0.0/0	1.9/2	0.0/0	2.6/22
Economic hardship								
Never	3.0/5***	0.3/1*	0.0/0*	0.8/2*	0.0/0	0.0/0***	2.4/1	0.6/9***
Quite often	15.1/36*	0.0/0*	2.0/4*	0.4/1*	0.0/0	2.0/3*	0.0/0	3.7/44*
Often	24.4/33	2.9/1	5.4/4	2.1/2	0.0/0	13.5/10	0.0/0	7.2/50
Always (R)	28.4/25	15.4/4	17.9/5	10.6/5	0.7/2	6.4/15	1.1/3	6.1/59

*n* number of observed persons, *R* reference group

*-*p* < 0.05, **-*p* < 0,01 and ***-*p* < 0,001 comparing with a reference group

A logistic regression was used to estimate the risk factors involved in the occurrence of refrain from buying prescribed medication due to cost (Table 4). The results revealed that being from Lithuania and experiencing economic hardship were related with increased risk to refrain from buying prescribed medications due to cost (OR = 14.92 and OR = 1.99 respectively).

**Table 4:** Logistic regression analysis of the relation between refrain from buying prescribed medication due to cost and selected socio-economic factors

Variable	OR	95 % CI	*P*
Being male	0.80	0.55-1.17	0.249
Age (each age group)	0.88	0.77-1.01	0.077
Education (higher level of education)	0.83	0.68-1.01	0.059
Living not alone	1.29	0.87-1.90	0.207
Being from Lithuania	14.92	10.33–21.56	*P* < 0.001
Daily worries about expenses (each group of more intensive worries)	1.99	1.68-2.35	*P* < 0.001

*p* significance level, *OR* odds ratio, *CI* confidence interval

## Discussion

Our study revealed that the prevalence of refrain was due to financial problems among older persons varied by country from 0.3 % in Spain to 15.7 % in Lithuania. Similar studies from other countries show variations by country as well. The reported underuse of medicines due to costs in older age varies from 3 % in the Australia, Canada, New Zealand, and the Netherlands to 8 % in Germany and 9 % in the United States [8].

How can these differences between ABUEL countries be explained? It is noteworthy that all countries which have participated in the study had pharmaceutical benefit schemes. This suggests that the financing of health-care systems can only partly explain differences in refraining from buying prescribed medications among older people. In spite of the presence of cost reimbursement mechanisms, patients need to make co-payments (or in some cases to pay the full price) for prescribed medications. In addition, studies indicate that chronic diseases require significant proportions of household incomes for buying medications [27]. This indicates that the purchasing power of people in the particular country can play a major role and be related with the economic situation in the country. Lithuania, which has reported the highest refrain rates, had the lowest gross domestic product (at the time of conducting this study) of all participating countries in the study [28]. It could be that economic factors influenced Lithuanians in deciding not to use prescriptions prescribed by their doctors. It is noteworthy that data for this study were collected during the economic crisis in Lithuania. In 2009, Lithuania experienced one of the highest annual decline of gross domestic product in the European Union (−14.8 %) [29]. This “*free fall*” of economy caused dramatic changes in salary policies, financing of health care and growth of unemployment [30]. It might be that “hard times” could have a negative impact on the economic accessibility to medications as well. On the other hand, it should be mentioned that the Lithuanian Government cut old-age pensions only from the year 2010 [31]. Therefore, we think that the infl uence of crisis on respondents’ answers is very limited. However, further investigations on this issue are needed. Moreover, the similar situation has been observed with accesibility to health care services within this age group during the period of economic recession in in Lithuania.

Our results show that the decision not to purchase medications was linked to individuals experiencing financial strain or problems. Similar patterns have been noticed in studies from Australia, Canada, New Zealand, United Kingdom and the United States [8].

We identified that females were more likely to refrain from buying prescribed medications due to costs. An explanation of this pattern could be that women in general report more bodily distress, and more numerous, intense and frequent somatic symptoms than men [32]. This may cause women to use more pharmaceutical products [20] and spend a considerable amount of the household income on them. It could be that women decided not to buy some of these prescribed medications in order to save financial resources. Correa-De-Araujo et al. (2005) showed that there are gender differences in use and expenditure on prescription drugs amongst older adults aged 65+. Overall, women spent about 17 % more than average expenditures by men. Several authors demonstrate evidence that it is critical for older women and men to have proper access to prescribed medicines, particularly women, given the financial vulnerability of the female population [33]. Moreover, Johnell and Parker (2011) state that women and men express themselves differently, report symptoms differently and probably also are treated differently – encounter different behavior – within the healthcare system. Socio-economic status may also play a part, since women, at least elderly women, often have a lower income and educational level than men. Socio-economic status may be linked to expectations, communication skills, how well informed you are about various treatments, what demands you place on healthcare and how you are treated within the healthcare system. It is therefore important to consider socio-economic status (e.g. education level) in analyses of gender differences, especially among elderly people [34].

The present study reveled inequalities in medication accessibility between different countries and within countries. It suggests a much broader plan of actions for solving this issue. Frost and Reich (2008) has developed a framework to describe the components of access to new health technologies, which can be adapted to accessibility to medications. According to the authors, there are three key components for improved access: availability (addressing both “upstream” issues of product discovery and development and “downstream” challenges of national pharmaceutical supply systems), affordability (sustainable funding and low prices), and safe and effective medicine use (rational use, quality and safety) [35]. These key components are affected by “architecture” of pharmaceutical system, which includes financing pharmaceutical workforce, governance, regulations etc. [36]. A complexity of this problem requires political support and sustainability. The recent WHO European policy framework and strategy for the 21st century “Health 2020” reminds about the commitment of WHO and its Member States to ensure universal coverage, including access to high-quality and affordable care and medicines and to eliminate catastrophic and impoverishing payments [37]. Some countries initiate specific activities to achieve these political objectives. Lithuania, which has demonstrated highest prevalence in cost-related non-adherence, has approved the “Lithuanian Health Program 2014–2025” in 2014 [38]. This national health policy emphasizes the importance in reducing health and health care (including accessibility to medications) inequalities in the country. There are several measures considered for this purpose but to name just few, there are: increase of public funding of health care system, strength of pharmaceutical care, use of health technology asseement in developing a list of state reimbursed medicines.

This study has some limitations. The participants (women and men) were recruited from urban centers in seven European countries and results might not be applicable to rural areas. Secondly, non-responders were not investigated. It could be, that those who refrained from buying prescribed medications were much higher among those who refused. Third, some confounding elements have been identified in the questionnaire. The main question of our paper is not asking, exclusively about medicines, but about care as well. This could cause, that answers reflect not only on medications. However, we expect only a minor effect of this factor, as the question is placed in the group of questions related to use of medications. This could lead, that respondents answering this question put more emphasis on mediations, rather other care. The absence of a specific time frame for refrain, could be identified as confounding factor as well. The fourth, the accuracy of the data was dependent on the participants’ subjective assessment of their situation. No objective evaluations (e.g. medical records) have been used to corroborate their responses. More detailed discussion on methodology and study limitations are published in a separate paper [24].

## Conclusions

The current study indicates that 3.6 % (*n* = 162) of older people refrained from buying prescribed medications due to cost. Living in Lithuania and financial strain were associated with self-reported underuse of medicines due to cost.

Non-adherence to medication prescribing occurs frequently in Europe and is associated with adverse outcomes and this impacts on patients, care providers and the healthcare system. As Ho et al. have emphasized: “*the first step toward improving adherence, there needs to be a broader recognition of the problem of non-adherence, and once identified, simple strategies should be implemented in daily practice to improve adherence*” [39].

## Footnotes

1 Greece excluded.
